# Teaching methods in Hawler College of Medicine in Iraq: A qualitative assessment from teachers' perspectives

**DOI:** 10.1186/1472-6920-12-59

**Published:** 2012-07-27

**Authors:** Abubakir M Saleh, Namir G Al-Tawil, Tariq S Al-Hadithi

**Affiliations:** 1 Department of Community Medicine, College of Medicine, Hawler Medical University, Erbil, Iraq

## Abstract

**Background:**

Medical education in Iraq is poorly assessed and there is a general lack of documented knowledge about the challenges facing this field and the needs for its development. This study aimed to assess the existing teaching methods in the Hawler College of Medicine, Iraq from teaching staff perspectives and assess the knowledge of the teaching staff about student-centred learning.

**Methods:**

A qualitative study based on a self-administered questionnaire survey of a purposive sample of 83 teaching staff in Hawler Medical University was conducted. The questionnaire addressed the participants’ view on the positive aspects and problems of the current teaching methods and priorities to change it. The qualitative data analysis comprised thematic analysis.

**Results:**

The study revealed significant problems facing the existing teaching methods including having large number of students in the lecture hall (45.0 %), having focus on teacher-centred teaching (45.0 %) and lack of infrastructures and facilities suitable for proper teaching (26.7 %). The priorities for improving the quality of teaching methods included adoption of small group teaching strategy in all study years (34.6 %), improving the infrastructure and facilities for teaching in the college (34.6 %) and provision of continuous academic development programs for the teaching staff (24.3 %).

**Conclusions:**

The existing medical education system face significant problems and it needs important and comprehensive improvements in different areas. There is a need for further research in this field to explore the identified problems in a more in-depth manner in order to better understand of the problems and needs of this important area of education.

## Background

Medical education is an important factor in progress of any country. The stage of progress in any country can be judged based on how they incorporate this field of education into their every day life and how this will affect the health care that is provided by health professionals [1].

The impact of teaching strategy used by the medical colleges play a major role in determining the effectiveness of their graduates. There are different teaching methods used by different medical colleges around the world; lectures, tutorials, seminars, class discussions, small group discussions, case studies, problem based learning, role playing, etc. Across the world, increasing attention is being given to the quality of teaching and learning in the medical colleges. There is also increasing pressure both to ensure effective teaching strategy and to incorporate innovate approach into medical curriculum to improve the skills and abilities of the graduates to meet the future challenges faced by them during their practical life [2,3].

Although traditional medical education methods had produced thousands of well-known, efficient and successful doctors in both developed and developing countries there are increasing calls for fundamental changes in medical education to meet the needs of the community. This is primarily due to innovative nature of the field and the strong social demand that future doctors should acquire sufficient interpersonal skills to deal with various patients. The manner of imparting medical education has been changing from a lecture-based, one-way approach to a more self directed manner of study [4,5].

Student-cantered learning (SCL) is currently attracting immense attention and is widely used in the teaching and learning literature. Many terms have been linked with student-centred learning, such as flexible learning, experiential learning, and self-directed learning and therefore the slightly overused term ‘student-centred learning’ can mean different things to different people [6,7].

Following establishment of several medical colleges in Iraq over the last few decades, medical students in their preclinical years are exposed to enormous amount of knowledge, primarily through lectures and readings, rarely supplemented by seminars [8,9]. However, during their clinical clerkships and residencies they switch to experiential learning as they begin to face the real problems of patients, an approach that differs fundamentally from what they acquire during their preclinical years [9,10].

The traditional lecture approach or the content-oriented approach is still the core teaching methods followed by Iraqi medical colleges. Several national activities have been adopted for reviewing medical college curriculum in Iraq over the last two decades. The purpose was to develop a national curriculum for medical colleges relevant to community needs [10].

Medical education in Iraq is poorly assessed and there is a general lack of documented knowledge about the challenges facing this field and the needs for its development.

This study aimed to assess the existing teaching methods in the Hawler College of Medicine, Iraq from teaching staff perspectives and assess the knowledge of the teaching staff about student-centred learning.

## Methods

This qualitative study was based on a self-administered questionnaire survey of teaching staff in Hawler Medical University in Iraq.

Hawler Medical University is located in Erbil city in the Iraqi Kurdistan Region. It includes of four colleges: Medicine, Dentistry, Pharmacy, and Nursing. Teaching in the four colleges is in English language. The University is affiliated to the Ministry of Higher Education and Scientific Research of the Kurdistan Regional Government. The College of Medicine was established in 1977 and is currently comprising of 12 different basic and clinical departments. It awards Bachelor degree in Medicine and Surgery (M.B.Ch.B).

The sampling strategy for selection of teaching staff to participate in this study involved stratification of the teaching staff according to different departments in the college and then according to their academic titles. Half of the teaching staff from each academic title stratum in each department were randomly selected and invited to participate in this study. In total 110 potential participants were recruited as key informants to participate in the study. The study was carried out during the period from 10^th^ April through 30^th^ October 2011.

A questionnaire was developed through literature review of curriculum assessments conducted in countries of similar contexts [11,12]. The questionnaire was developed in English language, which is the instructional language in the university . The questionnaire was pilot tested and subjected to two cycles of modifications based on iterative feedback received from 10 teaching staff.

The questionnaire comprised 5 closed items on age, gender, educational background, academic title, and years of teaching experience within the higher education. It also addressed the participants’ view on the positive aspects within the current teaching methods, and problems facing the methods, recommendations for improving the quality of the teaching methods and their knowledge about student-centred learning.

The qualitative data analysis comprised thematic analysis of open-ended questions using common coding techniques through reading the answers on the questions and identifying main themes within the answers. Using these identified themes, a structured classification of codes was generated. The data were coded in a series of iterative steps, and the code structure was revised and refined multiple times as new insights were developed and new relationships between the themes present in the answers were elicited. Each open item was analyzed and reported independently except for items of education and training needs that were analyzed and reported with the item of priority needs.

More than one response was coded for each subject when necessary. Duplicate answers were only coded once. Illegible, blank, and off-subject answers were coded as missing data. Great care has been taken to preserve the anonymity of survey participants and their institutions.

The Research Ethics Committee of Hawler Medical University study approved the study and an informed consent was obtained from each participant after giving them full information about the study; explaining to them the aim of the study; that the participation is voluntary and no money or other incentive would be given to participations, The risk from participating in the research; that the information they provide will not be divulged to others without their permission, and that their identities will not be disclosed to third parties.

## Results

Out of the 110 teaching staff invited to participate in the survey, 83 staff completed the questionnaire giving a response rate of 75.4 %. The mean age ± SD of the respondents was 43.8 ± 8.1 years with an average experiences in higher education of 7.1 ±6.2 years. Other demographic and professional characteristics of the respondents are shown in Table 1.

**Table 1:** Demographic and professional characteristics of the respondents (n = 83)

**Characteristic**	**No.**	**(%)**
**Gender**		
Male	58	(69.87)
Female	25	(30.12)
**Academic title**		
Professor	2	(2.40)
Assistant professor	17	(20.48)
Lecturer	40	(48.19)
Assistant Lecturer	24	(28.91)
**Educational background**		
M.Sc & Higher Diploma	28	(33.73)
PhD, Iraqi and Arab Board, Membership of the Royal Colleges of the U.K & equivalent degrees	55	(66.26)
**Specilities**		
Basic science	29	(34.93)
Clinical science	54	(65.06)
**Place of graduation**	15	(18.07)
Iraq	74	(89.15)
Abroad	9	(10.84)

The majority of respondents agreed that the current teaching method in the college is teacher-centred rather than student centred and that students have no role in the process (86.7 % and 84.3 %, respectively).

Fourty nine respondents (59.1 %) recognized a positive attitude toward existing teaching methods particularly in using of different teaching aids by teachers in the classroom (57.1 %), adoption of small group teaching (46.9 %), participation of students in discussion in the classroom (20.4 %), distribution of handout to the students (6.1 %).

Seventy one respondents (85.5 %) identified certain problems in the current teaching methods particularly the large number of students in the lecture hall (45.0 %); teacher-centred teaching method (45.0 %); lack of facilities and infrastructures suitable for proper teaching (26.7 %); inadequate practical and clinical sessions which was attributed to limited spaces in hospitals and unavailability of cases (15.4 %); lack of interest and motivation of most of students to participate in discussion in the classroom (15.4 %); some of the teaching staff are not well trained with poor scientific background and language in addition to inappropriate behavior (11.26 %). Details of positive aspects and problems facing the teaching methods are shown in Table 2.

**Table 2:** Positive aspects and problems identified by teaching staff (n = 83)

**Variable**		**Response**	
		**No.**	**(%)**
**Positive aspects (n = 49)**			
1	Using of different teaching aids in the classroom	28	(57.1)
2	Adoption of small group teaching	23	(46.9)
3	Participation of students in the discussion in the classroom	10	(20.4)
4	Distribution of handout to the students	3	(6.1)
5	Implementing OSCE examination in some subjects	2	(4.0)
6	Implementing mid & final year examinations	2	(4.0)
7	Receiving feedback from students	2	(4.0)
**Problems (n = 71)**			
1	Large number of students in the lecture hall	32	(45.0)
2	Teacher-centred teaching	32	(45.0)
3	Lack of infrastructure and facilities suitable for proper teaching	19	(26.7)
4	Inadequate practical and clinical sessions	11	(15.4)
5	Most of the students are not interested & not participating in discussions	11	(15.4)
6	Some of teaching staff are not well trained with poor attitude, scientific background and language in addition to inappropriate behavior	8	(11.2)
7	Bad teacher-student relationship	5	(7.0)

Seventy eight respondents (93.9 %) have recommended a number of priorities for improving the quality of the teaching methods in the college like: implementing small group teaching in all study years (34.6 %); improving the infrastructure and facilities for teaching (34.6 %); continuous training of the teaching staff about updated methods of teaching in higher education institutes (24.3 %); giving more active role for students in the learning process (23.0 %) and focusing more on practical and clinical sessions (16.6 %). Details of the recommendations for the improving the teaching methods as identified by the respondents are shown in Table 3.

**Table 3:** Priorities for improving the quality of teaching methods as identified by the respondents

**Priority areas**		**Response**	
		**No.**	**(%)**
1	Implementing small group teaching in all study years.	27	(34.6)
2	Improving infrastructure and teaching facilities.	27	(34.6)
3	Continuous training of the teaching staff about updated methods of teaching.	19	(24.3)
4	Giving more active role for students in the learning process	18	(23.0)
5	Focusing more on practical and clinical sessions	13	(16.6)
6	Building better student-teacher relationship	8	(10.2)
7	Changing the teaching into block system	6	(7.6)
8	Use more teaching aids and skill lab in the learning process	6	(7.6)
9	Regular feedback from students	4	(5.1)
10	Getting use of the experiences of other medical colleges of neighbouring countries	4	(5.1)
11	Increasing number of staff in some departments	3	(3.8)
12	Revising the examination system	2	(2.5)
13	Improving cooperation between different departments in the college	2	(2.5)

The majority of respondents were moderately satisfied with the current teaching methods (strongly satisfied: 10.8 %, moderately satisfied: 57.8 %, poorly satisfied: 22.9 %, unsatisfied: 8.4 %).

Fourty three respondents (51.8 %) have heared about student-centred learning approach with inadequate different view of the term. Different definitions were given by the respondents including: interactive sessions in which students are active and participate in the discussions (44.1 %); active participation of students in the whole learning process including curriculum design,teaching methods, and assessment (16.2 %); a teaching method that focus on the needs, ability and interest of the students (11.6 %); a small group teaching (11.6 %); shifting the power and focus of activity from the teacher to students (6.9 %); student have full responsibility for their learning (6.9 %) and problem based learning (2.3 %) (Table 4).

**Table 4:** Correspondent's definition of student-centred learning

**Definition of student-centred learning**		**Response**	
		**No.**	**(%)**
1	Interactive sessions in which students are active and participate in the discussions.	19	(44.1)
2	Active participation of students in the whole learning process including curriculum design,teaching methods,and assessment	7	(16.2)
3	A teaching method that focus on the needs, ability and interest of the students.	5	(11.6)
4	A small group teaching	5	(11.6)
5	Shifting the power and focus of activity from the teacher to the students.	3	(6.9)
6	Student have full responsibility for their learning	3	(6.9)
7	Problem-based learning	1	(2.3)

Twenty one out of 43 (48.8 %) respondents who have heared about student-centred learning considered the teaching methods they use like clinical sessions (38.0 %), practical sessions (33.3 %), seminars (14.2 %) and small group teaching (14.2 %) as student-centred approaches (Table 5).

**Table 5:** Examples of current teaching methods described by respondents as SCL

**Examples**		**Response**	
		**No.**	**(%)**
1	Clinical sessions	8	(38.0)
2	Practical sessions	7	(33.3)
3	Seminars	3	(14.2)
4	Small group teaching	3	(14.2)

## Discussion

This study revealed that the teaching method in the college is largely teacher-centred (content-oriented) rather than student-centred (learning-oriented) with very limited role for students in the teaching process. Iraqi medical colleges in general follow traditional teacher-centred curriculum since the establishment of the first medical college in Iraq in 1927 [11]. Undergraduate medical curriculum in Hawler Medical College is not much different from that of the rest of Iraq. Few changes have been adopted by the college like introducing small group teaching in 5^th^ year since 2007 to meet increasing complexity of medical education and to prepare more qualified doctors in the future. Although implementing small group teaching in the 5^th^ year was a good step but it was not well planned and adequate infrastructure was not available for successful implementation of this method of teaching.

Use of different teaching aids by teachers in the classroom, having a small group teaching, participation of students in discussion in the classroom were recognized as the main positive aspects of the current teaching system. Use of audio-visual teaching aids in the classroom is widely used in the college and it is well perceived by the students. Small group teaching is an important component of undergraduate medical education; many medical schools around the world have adopted this strategy of teaching to make the classes more interactive and to give opportunities for students to take part in discussion [13,14].

Many negative aspects or problems were reported about the current teaching methods including large number of students in the lecture hall, teacher-centred curriculum, lack of facilities and infrastructures suitable for proper teaching. Admission to medical colleges in Iraq is solely based on high school marks, in which students with the highest marks were admitted. In Kurdistan Region there are only three medical colleges and there is rapid increasing population in the region due to many factors including that the region is secure in comparison to the other parts on Iraq [15].

Different themes were reported as priorities to improve the quality of teaching methods particulary application of small group teaching in all years of study in the college, improving the infrastructure and teaching facilities, continuous training of the teaching staff about updated methods of teaching, changing current teaching methods into more interactive giving more active role for students in the learning process. Due to lack of suitable infrastructure and facilities, small group teaching is only implemented in 5^th^ year and on small scale in other years of study like practical sessions in preclinical years and clinical sessions in clinical years.

While many positive aspects and problems facing teaching methods as well as recommendations mentioned by the participants of this study are similar to the finding of other studies done in other countries, there are some new problems and recommendations emerged from the results of this study. Problems like depending on lectures and other didactic methods where the activity is teacher–centred were also reported by studies conducted in other countries like Saudi Arabia [16] and Iran [17] and the problem of inadequate number and poorly trained teaching staff was reported by studies from Libya [1] and Saudi Arabia [18]. Lack of suitable infrastructure and facilities for teaching was reported also in Saudi Arabia [16] and Iran [17] and the problem of large number of students reported in Iran study [17]. New problems emerged from this study included poor teacher-student relationship, inadequate practical and clinical sessions, poor interest and participation of the students in the classroom.

The priorities to changing the curriculum toward more student-centred were also reported by other studies from Libya [1], Saudi Arabia [16,18] and Iran [17]. Priorities for increasing number and training of teaching staff were also reported by studies from Libya [1], Iran [17] and Saudi Arabia [18]. Improving the infrastructure and facilities to be suitable for proper teaching was reported by a study from Saudi Arabia [16]. Other priority needs emerged from this study included more focus on practical and clinical sessions, building better relationship between teacher and students, using more teaching aids and skill laboratories in the learning process, regular feedback from students, getting use of the experience of other medical colleges of neighbouring countries, revising the examination system and improve cooperation between different departments in the college.

More than half of the respondents (51.8 %) have heared about student-centred learning approach and those were unsure as to what the term meant. This finding is a positive point since the research was conducted at a university which did not adopt the student-centred learning approach as learning and teaching strategy on large scale yet.

Seven different definitions of student-centred learning emerged from the data. Most teaching staff (44.1 %) held the following definition:

*"Interactive sessions in which students are active and participate in discussion in the classroom".* We anticipated this difficulty in examining teaching staff belief about student-centred learning because it is a broad term and have been described differently by different authors [6,7,19,20].

Some of the teaching staff described their own clinical teaching (38.0 %) and practical sessions (33.3 %) as student -centred learning. Indeed, they viewed any activity in which students are active and working in group with their classmate as student-centred.

The use of qualitative approach with open-ended questions in this research involves several limitations that require consideration. Because qualitative research involves complex analysis and interpretation of the issue under investigation, this makes it difficult to be conducted in areas with little experience about this type of research like Iraq [21]. In open-ended questions, respondents usually face problems in completing the questions being of different level of education, background, and teaching experience [22].

Since this study has only involved Hawler College of Medicine, its findings might not be generalizable to other universities in Iraq. However, based on our knowledge we think that the general situation of the teaching methods in the other universities in Iraq might be similar to a large extent.

## Conclusion

The existing medical education system face significant problems and it needs important and comprehensive improvements in different areas. There is a need for further research in this field to explore the identified problems in more in-depth manners in order to better understand of the problems and needs of this important area of education.

## Abbreviations

M.B.Ch.B, Bachelor degree in Medicine and Surgery.

## Competing interests

The authors declare that they have no competing interests.

## Authors’ contributions

AMS and NGT conceptualized the study. NGT, TSAH and AMS participated in designing the study. NGT and AMS supervised data collection and carried out data analysis. AMS and NGT drafted and finalized the manuscript. TSAH extensively reviewed and edited the manuscript. All authors contributed to interpreting study results and writing the manuscript. All authors read and approved the final manuscript.

## Pre-publication history

The pre-publication history for this paper can be accessed here:

http://www.biomedcentral.com/1472-6920/12/59/prepub
